# Bacteriology of the Mouth

**Published:** 1900-03-15

**Authors:** L. T. Ivins


### BACTERIOLOGY OF THE MOUTH.

BY DR. L. T. IVINS.

Read before the Cincinnati Odontological Society, December 29, 1899.

Bacteria belong to the group of fungi; they are unicellular plants with an astonishing capacity for rapid reproduction by division, when placed under favorable conditions of warmth and moisture; some requiring oxygen, others being independent of it, or, at all events, independent of air. These latter are termed anaerobic.

A large majority of bacteria are saprophytes, that is to say, they prey upon dead and decomposing vegetable or animal matter, it being supposed that their protoplasm acts as a ferment, and so sets up chemical actions which result in the splitting up and re-arrangement of the organic compounds about them

Bacteria attack both living and dead bodies, and, by their reproduction in the former, cause a great many serious and fatal diseases

The bacteria which cause these effects are termed pathogenic. As the mouth presents all the conditions necessary in the way of warmth, food and moisture, it is not to be wondered at that a very large number of different bacteria have been met with in it, and it is more than probable that cleanliness of the mouth, and as near an approach to an aseptic condition as is attainable under very difficult condititions which it presents, may have very much more effect in the warding off of serious diseases than most people at all realize.

Those bacteria which are of rod-shape are usually called bacilli, while those which are round are called cocci. Both are frequently aggregated into chains and in that form are called streptococci, or into cluster or packets then they are known as staphylococci.

Miller has obtained and cultivated upward of a hundred different forms from the human mouth, pathogenic and non-pathogenic; among them, the micrococcus of sputum septicæmia, the staphyloccocus pyogenes, one of the most frequent causes of suppuration, the pneumococcus the cause of pneumonia, and many others.

There are, however, some six forms which are to be found in almost every mouth, and it happens that all of these present the peculiarity that no culture medium has as yet been found upon which they will grow. These are: Lepthothrix innominata, bacillus buccalis maximus, leptothrix buccalis maxima, iodococcus vaginatus, spirillum sputigenum, spirochaete dentium.

Besides these organisms there is a vast number of others which are present at times, and which are pathogenic, so far at least as animals are concerned. Miller estimated that in one very unclean mouth there were not less than one thousand one hundred and forty millions of organisms.

It may, therefore, be easily understood that human saliva is at times toxic, sometimes very virulently toxic. so that animals inoculated with it die within thirty hours.

In cases of pyorrhea a microccocus is found, which can be cultivated, and which causes abscess and sometimes death in small mammals. It may be regarded as certain that the disastrous results which occasionally ensue after wounds of the mouth in tooth extraction, which in a not inconsiderable number of cases have resulted in death of the patient, are due to infection with micro-organisms of virulently pathogenic powers which have existed in the mouth. And while we can not be too careful that our instruments are not the source of the introduction of a poison, yet it is probable that in many cases which have been ascribed to dirty instruments the organism has really been present in the mouth and has merely been carried deep into the tissues by the operation.

There is proof that pathogenic bacteria in the mouth may find their way into the digestive system or into the lungs.

Streptococci are frequently pathogenic, that is, are the active causes of diseases ; in scarlet fever they are often found in the tonsils and may cause general septicæmia or pyæmic infections, erysipelas, abscesses, purulent peritonitis, ulcerative endocarditis and other forms of disease.

Bacteria must be constantly passing into the stomach with food. Some of these perish in the gastric juice and *some* do not.

Bacteria cause fermentation and putrefaction, and those fermentations with which we are most concerned are such as result in the formation of acids by the transformation of carbohydrates into lactose and lactic acid.

A large number of bacteria are capable of effecting this change, although there is one particular one which has been met with in the mouth which has such a decided power in this direction that it has been called the bacterium acidi lactici.

Other fermentations beside this most important one take place. A gummy product is sometimes formed, and is believed by Black that this is the cause of sordes upon the teeth in fever, and that it may be a cause of stringy saliva.

The difficulty of securing aseptic conditions in the mouth is so great as to, in many cases, render them impossible to attain, although Dr. Miller has shown that by the assiduous use of antiseptic washes, the use of floss-silk, etc., the number of organisms is greatly diminished. Mercuric chloride in the strength of 1 to 2,500 was, according to his experiments, the most efficacious.

Many dentists, in operations upon the mouth, knowing complete asepsis impossible, are less careful than they would consider right elsewhere in the body.

This should not be so. If we can only diminish the number of active bacteria which come in contact with the wounded surface, the gain is worth the trouble

If we use instruments and fingers which are possibly not clean, we may introduce some specially pathogenic organisms which were not there beforehand, though swarms of non-pathogenic may have been.

Hence, when any operation in the mouth is contemplated, it and the instruments to be used should be rendered aseptic before commencing the operation.

DISCUSSION OF DR. L. T. IVINS' PAPER.

Dr. Frank Hunter: The question of the importance of the influence of bacteria in the mouth is, as I understand it, still an open one. I have been for years a stickler for cleanliness, perfect cleanliness in the dentist's office, for nicety in the care ot dental instruments to be used about the mouth. Bacteria we know exists in all parts of the body. They are found in the food we eat, in the water we drink; the air we breathe is full of germs. Deprive the air of its bacterial germs and we die. I have doubted whether boiling water is a good thing. We not only destroy the pathogenic germs by boiling, but the germs which give life to the water, also. It may be advisable to boil the water which is derived from streams, which being low in summer contain in a concentrated form impurities which at other seasons are diluted and practically innocuous, owing to a higher stage of water.

It is, at all events, a difficult matter to sterilize the human mouth. The various preparations recommended as mouth washes are very pleasant to use, but aside from the office of cleansing, they are of doubtful utility as a means of destroying germs. The sterilization of instruments is all good and well. Many dentists are slovenly, as regards this matter. Some go to extremes in the other direction. I know a prominent Boston dentist who sterilizes not only his instruments, but claims to put even the coat he wears while operating into the sterilizer. However, he does not sterilize his hands.

We know that bacteria are present in decay of the teeth, but we do not know that they are the cause of decay. I confess to a feeling of reluctance to abandon wholly the chemical theory of decay propounded by Dr. George Watt, years ago. I do not deny the truth of Dr. Miller's conclusions that germs are the sole cause; I merely say of that theory that it is not proven. My private opinion is that in a combination of the theories of these two investigators we will find nearly the truth.

Dr. Kumler: The question arises in my mind, after we have sterilized our instruments and when we come to use them in a patient's mouth, are they not liable immediately to become infected with the poison which may be present in that patient's mouth, and may we not at once poison that patient if by chance we wound a membrane?

Dr. Heise: I would not have the members misunderstand Dr. Hunter. He believes in bacteriology; no doubt of that. I do not believe the man lives who would deny the truth of that science. Whether or not bacteria do the harm that bacteriologists claim they do may be a question. We dentists do not inflict on the soft tissues of our patients the same injuries that the surgeon inflicts and therefore our ministrations to them are less likely to be followed with disastrous consequences, in case even we neglect precautions in sterilizing. That however is purely incidental, and does not at all justify neglect of precautions, since it may happen at any moment that we will inflict upon a soft tissue some wound favoring the ingress of germs. If we attempted to perform on our patients such operations as the surgeon performs, without taking such antiseptic precautions as they take, we should kill our patients.

Dr. Frank Sage: We often have repeated to us the precaution familiar to us all, touching the necessity of using sterilized broaches in roots. Now, without denying that micro-organisms may be the chief agents causing inflammation in those cases where the dentist incautiously forces effete matter through the foramen of a tooth, I call attention to a statement of Dr. Austin Flint, who refers to pressure as one of the causes of inflammation in general. Applying this suggestion to the abscessed tooth under treatment, what conclusion may we draw from the fact that often the dentist winding cotton about a broach, pumps medicaments through the root-foramen into the tissues beyond, thus exerting the force of a piston on the already inflamed tissues, and so aggravating the inflamed condition? Is it simply that he has helped the organisms to a position favorable to exercising their specific function on a part before inaccessible? Or has the simple fact of pressure exerted on the already inflamed tissue any significance in accounting for an exacerbation of the symptoms?

Many men eminent in the medical profession, men who reason and who are earnest seekers after truth, hold greatly modified views of the germ theory. They admit so much of the truth of the theory as to acquiesce in the demand for extreme cleanliness in the care of all instruments and appliances, and for personal cleanliness on the part of the operator, when serving a patient in any surgical capacity.

in particular. For my own part, I have wondered that in all that has been said and written on the subject of sterilizing instruments I do not recall any reference whatever to the need of sterilizing the utensils and implements of the dental laboratory. The dentist uses the same wheels in his lathe for brushing a new plate and any old repaired plate; he uses the same scrapers, the same grits and polishing powders, the same clamps, sponges, burnishers. If this danger of infection be so ever-present, why neglect precautions in our laboratories?

I confess that suggestions which have been made as to means to be employed to effect perfect sterilization of the hands of the dentist before operating have seemed to me grotesque to the point of being absurd. Dr. Miller, for instance, years ago wrote that to effect this purpose the hands should be washed for fifteen minutes in soft soap, this to be followed by a further laving for fifteen minutes in a weak carbolic acid solution. It may be that Dr. Miller enjoys a reputation as a wag, of which I have not been apprised. At all events, I am inclined to regard this suggestion as of the nature of satire. How many times a day would consistency require of the busy dentist a repetition of this procedure?

If the danger of infection is so great why is it that we do not hear of frequent cases of disease of an infectious character, resulting from eating at public restaurants? Cleanliness is not always a prime consideration at eating-houses.

I use care in thoroughly cleansing all instruments employed in daily practice. Hot water, boiling hot, soap and a stiff brush are my chief reliance. I regard that as all-sufficient, by way of precaution against infection of my patients. I have never but once had a case of infection laid at my door in more than a quarter of a century of practice, and even that case—it was a rather virulent case of blood poisoning—*dental* poisoning, the country doctor somewhat vaguely named it—I attributed later to a big blue-bottle fly hovering about my patient's favorite fever blister, carrying contagion from the cuspidor, I surmised.

Dr. Heise: The great danger of infection comes from any existing lesion in a mucous tissue or from any accidental wounding of such tissue. Pathogenic germs may be present in the mouth doing no injury until you perhaps wound a part, when they find ingress to a field where they do mischief. As the mucous tissue is usually well protected by epithelium, these germs are excluded from regions where otherwise they would be absorbed. If for no other reason I hold that the dentist owes it to his patient to use extreme precautions in sterilizing his instruments, because of the danger of communicating syphilis, which disease is far more prevalent than many dentists suspect, and that in the cases of patients innocent of immoral practices. Ladies are often treated for this disease without the real nature of the disease being made known to them.

That pathogenic germs may exist in the mouth without evincing their presence until some action on our part calls them into activity, is proved by the fact that sometimes, even in so simple a matter as removing tartar, the instruments being known to have been thoroughly sterilized, I have seen severe inflammations and even necrosis follow. I do not say that the necrosis was extensive enough to involve the entire jaw, but it did exist.

Dr. Grant Molyneaux: Do I understand you to say that you believe necrosis to have resulted from infection with the patient's own germs, owing to wounding of the mucous membrane while removing tartar?

Dr Heise: Yes, sir.

Dr. Molyneaux: That I do not believe.

Dr. Sweeny: We sterilize instruments and put them away in drawers, and there, so far as I can make out, they are just as much liable to infection as ever. The germs are all about us, in the air. I have observed the necessity of taking certain precautions against infection in cases where I have found it very particularly required. Thus, for instance, after extracting a lower molar, badly broken down by decay, with accompanying inflammation, I take pains to wash out the socket with hot water, using also antiseptics, to avoid the danger of absorption of some pathogenic germs floating in the mouth. In two cases I might refer to, infection was obviously from germs already in the patient's mouth, not from an unsterilized instrument.

DR. MOLYNEAUX: Sir Lawson Tait never made a boast about using antiseptics of any kind. He constantly urged cleanliness, cleanliness, cleanliness! We are in no danger of infecting our patients until we will say, for instance, a bur slips and wounds a tissue; then there *is* danger. If you push with a broach on and on into a root, you finally reach soft tissue and cause infection.

After extraction of a very sore tooth, if you cauterize the socket with nitrate of silver or any cauterant you are almost sure to aggravate the trouble, through closing in the pathogenic germs. Better to wash out with warm water, and, if necessary, to use a mild antiseptic; at all events, do not cauterize. You might as well have the offending tooth there. Free drainage is the great requisite

The contagion of any virulent poison is not so much to be feared unless you puncture the soft tissue with a broach or instrument not rendered aseptic. There the real danger lies.

DR. ROSE: Dr. Conner says that half a drop of pus from any mouth, taken into the stomach, is liable to cause blood poisoning And yet we find persons going about week after week and month after month with fistulae discharging into their mouths, and no blood poisoning.

Dr. Heise: In those cases there is no specific germ at hand to infect the individual, or the germ may be present and the patient for the time being is immune. But let that patient become run down in some way, the tone or vitality decline slightly or considerably, and then the germ may become operative, where before it was wholly innocuous. That is a liability to which all are exposed at times.

